# Antibacterial activities of anthraquinones: structure–activity relationships and action mechanisms

**DOI:** 10.1039/d3md00116d

**Published:** 2023-07-10

**Authors:** Tang Qun, Tiantian Zhou, Jiongkai Hao, Chunmei Wang, Keyu Zhang, Jing Xu, Xiaoyang Wang, Wen Zhou

**Affiliations:** a Shanghai Veterinary Research Institute, Chinese Academy of Agricultural Sciences 200241 Shanghai China wxy@shvri.ac.cn zhouwen60@126.com; b School of Chinese Materia Medica, Guangdong Pharmaceutical University 440113 Guangzhou China; c Key laboratory of Veterinary Chemical Drugs and Pharmaceutics, Ministry of Agriculture and Rural Affairs, Shanghai Research Institute, Chinese Academy of Agricultural Sciences Shanghai 200241 China; d Huanghua Agricultural and Rural Development Bureau Bohai New Area 061100 Hebei China

## Abstract

With the increasing prevalence of untreatable infections caused by antibiotic-resistant bacteria, the discovery of new drugs from natural products has become a hot research topic. The antibacterial activity of anthraquinones widely distributed in traditional Chinese medicine has attracted much attention. Herein, the structure and activity relationships (SARs) of anthraquinones as bacteriostatic agents are reviewed and elucidated. The substituents of anthraquinone and its derivatives are closely related to their antibacterial activities. The stronger the polarity of anthraquinone substituents is, the more potent the antibacterial effects appear. The presence of hydroxyl groups is not necessary for the antibacterial activity of hydroxyanthraquinone derivatives. Substitution of di-isopentenyl groups can improve the antibacterial activity of anthraquinone derivatives. The rigid plane structure of anthraquinone lowers its water solubility and results in the reduced activity. Meanwhile, the antibacterial mechanisms of anthraquinone and its analogs are explored, mainly including biofilm formation inhibition, destruction of the cell wall, endotoxin inhibition, inhibition of nucleic acid and protein synthesis, and blockage of energy metabolism and other substances.

## Abstract 2

This review article systematically summarizes the structure–activity relationships of anthraquinones and sheds light on their antibacterial mechanisms.

## Graphical abstract

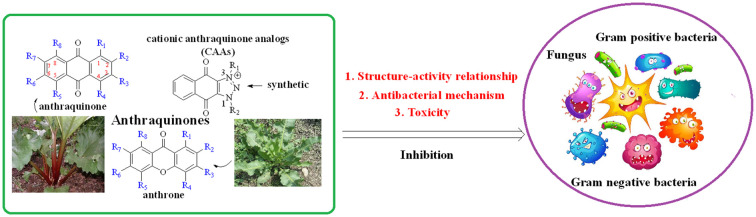

## 1. Introduction

Currently, clinicians are still treating bacteria or fungi causing infections with antibiotics;^1,2^ however, antibiotics have many serious adverse effects.^3,4^ Antibiotics are exerting antibacterial efficacy, while pathogenic bacteria are constantly adapting to them, resulting in drug resistance. Coupled with the misuse of drugs, the failure of existing antibiotics leads to the subsequent creation of some superbugs.^5,6^ The emergence of multidrug-resistant bacteria and antimicrobial resistance genes has become a global medical challenge, which threatens human health and causes huge economic losses to livestock breeding and other industries.^7–9^ Accordingly, a search for new drugs to combat multidrug-resistant bacteria is highly urgent.^10,11^ Throughout the history of the discovery of many antimicrobials, natural products as a main source, such as quinones, coumarins, flavonoids, macrolides, xanthones, *etc.*, have attracted the attention of many scholarsapos due to their low toxicity, wide distribution and high activity, which are highly valuable for research and development.^12,13^

Anthraquinone and its derivatives are widely distributed in nature, *e.g.* plants and some microorganisms.^14,15^ Anthraquinones including aloe rhodopsin, aloe glycosides, and rhodo are the active ingredients of many Chinese herbal medicines such as *Rheum palmatum* L., *Fallopia multiflora* Harald, and *Reynoutria japonica* Houtt.^16,17^ Aloe emodin displayed anti-inflammatory, anti-tumor and antibacterial activities.^18–20^ Structurally, aloin similar to aloe emodin has antibacterial activity along with neuroprotective and nephroprotective effects.^21–23^ In addition to antibacterial activities, anthraquinones exhibit antitumor and antidiabetic activities,^24,25^ highlighting that they are extremely valuable for medicinal research and development. Meanwhile, the marketed anthraquinone antibiotics, such as fluoroquinolones, have a wide antibacterial spectrum, strong antibacterial activity, and stable chemical properties. The broad-spectrum and potent antibacterial activity of tetracycline, the most widely used synthetic antibiotic in the world, has enormously contributed to controlling bacterial infection. In recent years, anthraquinone derivatives with an adjustable activity and selectivity have emerged, and their major advantages are unique: (1) low production cost; (2) the accessibility of diverse structural variations with biologically relevant parts; (3) drug resistance is hard to develop, providing a novel direction for developing selective antibacterial drugs.

**Fig. 1:**
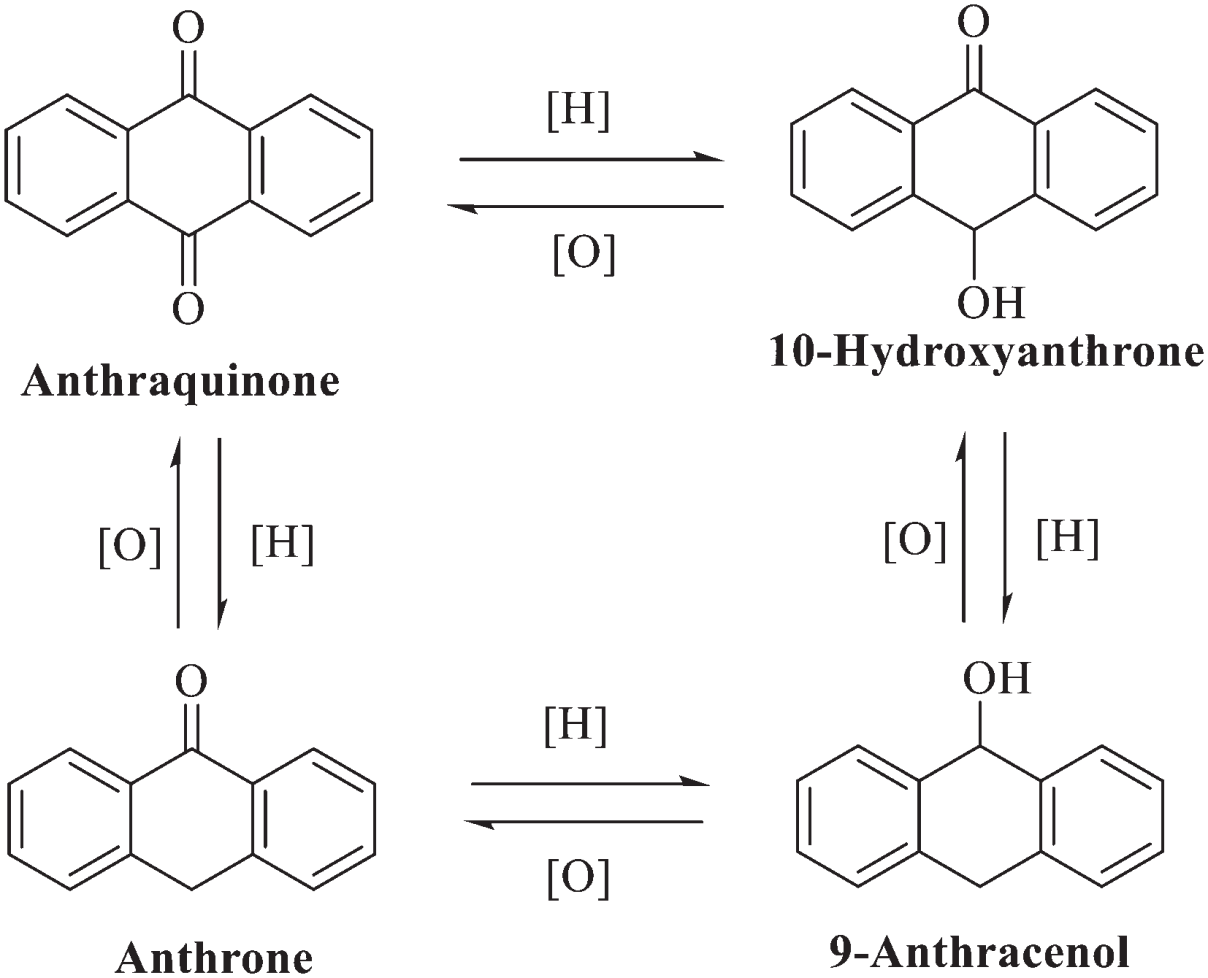
Interconversion of the anthraquinone parent nucleus with different redox degrees.

**Fig. 2:**
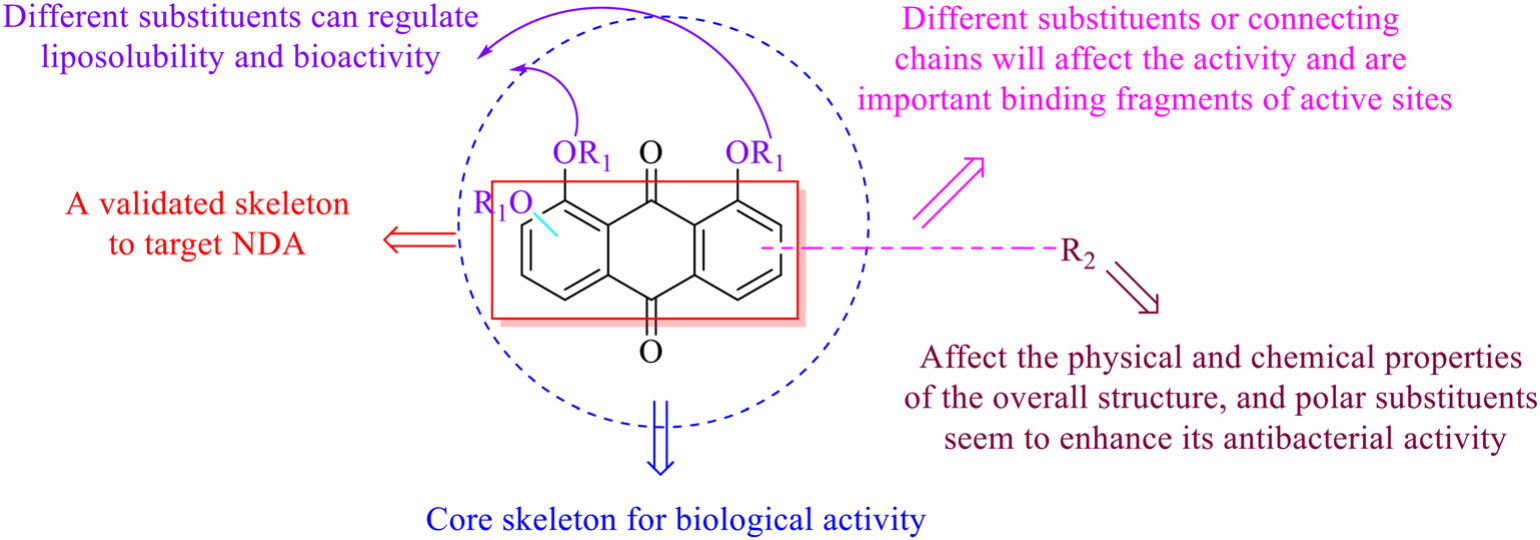
The structure–activity relationships of anthraquinones.

Anthraquinone is a kind of polycyclic compound with a quinone structure, that is, it contains a cyclohexadiene diketone or cyclohexadiene dimethyl structure. Its parent nucleus is 9,10-anthraquinone, on which there are methyl, hydroxyl, carboxyl, methoxy and amino substituents. Anthraquinone-type compounds include anthraquinone derivatives and reduction products of different degrees. Anthraquinone is generally divided into the alizarin type and rhein type.^24^ According to the degree of reduction, anthraquinone can be further divided into three categories: anthraquinone derivatives, anthrone derivatives and anthracenol derivatives (Fig. 1). In terms of anthraquinone parent cores, anthraquinones can be categorized into single anthraquinone, double anthraquinone and anthraquinone glycoside.^25,26^ With the discovery of more and more plant extracts with antimicrobial activity, anthraquinones identified hold greater potential in developing antimicrobial agents. Although some reviews have documented the pharmacological activity of anthraquinones, few reports are provided regarding their structure and antibacterial activity relationships and action mechanisms.^27,28^ Therefore, in this review, we will firstly introduce the relationship of the structure and activity of anthraquinone derivatives according to different classifications of anthraquinones, summarized in Fig. 2 and 4–6, and then the antibacterial mechanisms and toxicity profiles of anthraquinones will be systematically discussed. Through the analysis and summary of the structure–activity relationships and antibacterial mechanisms, we hope to provide a meaningful guideline for designing and finding more efficient, low-toxicity and safer anthraquinone-based antibacterial agents.

## 2. Structure and antibacterial activity relationships of anthraquinones

### 2.1 Monoanthracene nuclei

#### 2.1.1 Anthraquinones

##### 2.1.1.1 Rhein-type anthraquinones

The hydroxyl groups in this kind of anthraquinone are distributed on the benzene rings of both sides. Emodin (1) was first discovered at the end of the 18th century. The physiological activity of emodin determines its use in medical treatment, health care and daily chemicals. The antibacterial activity of emodin has been shown to have broad-spectrum antibacterial activity,^29^ such as against *B. subtilis* and *S. aureus*.^30^ It was also confirmed by experiment^31^ that, superior to that of the control oxacillin (MIC = 128 μg mL^−1^), emodin from *tigrinum* exhibited significant anti-MRSA252 activity (MIC = 4 μg mL^−1^), and another experiment^32^ that 0.5–2.0 mg mL^−1^ emodin inhibited the *in vitro* activity of *Streptococcus mutans* and prevented it from secreting acidic substances and synthesizing glucan. The inhibitory effect of emodin on *Helicobacter pylori* strains SS1 and ATCC 43504 (ref. 33) was observed and an action target to inhibit *Helicobacter pylori* β-hydroxyacyl ACP dehydratase was found. Numerous reports on the structures and its antibacterial activity of emodin and its derivatives are listed in Tables 1 and 2, respectively, and some conclusions are reached that the broad-spectrum antibacterial effects of emodin were achieved by interacting with biofilms, interfering with DNA or protein synthesis, or inhibiting other substances.

**Table 1:** Structures of anthraquinones

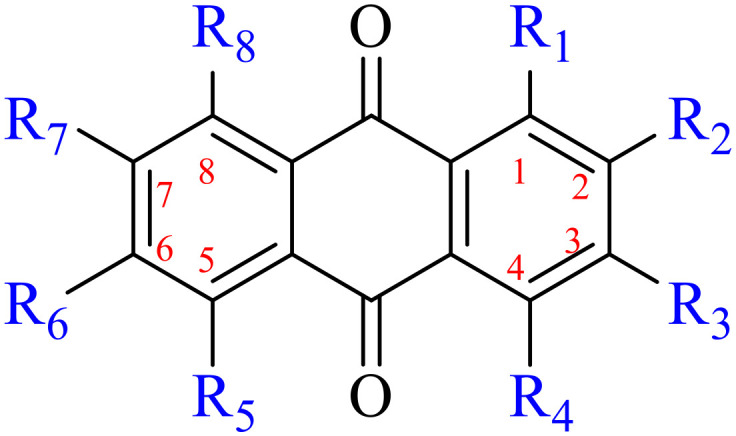								
Name	R_1_	R_2_	R_3_	R_4_	R_5_	R_6_	R_7_	R_8_
1	OH	H	OH	H	H	CH_3_	H	OH
2	OH	H	CH_3_	H	H	H	H	OH
3	OH	H	COOH	H	H	H	H	OH
4	OH	H	CH_2_OH	H	H	H	H	OH
5	OH	H	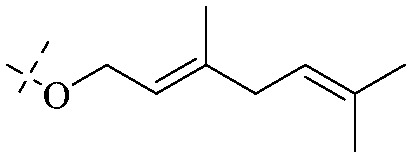	H	H	CH_3_	H	OH
6	OH	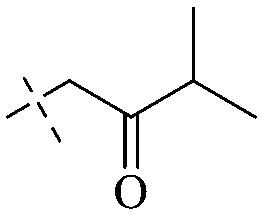	OCH_3_	H	H	H	H	OH
7	OH	H	OCH_3_	H	H	H	H	OH
8	OH	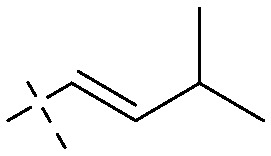	OCH_3_	H	H	H	H	OH
9	OH	H	CH_3_	H	Cl	H	H	OH
10	OH	H	OCH_3_	H	H	CH_3_	OH	OH
11**HEI1**	OH	H	CH_3_	H	H	CH3	I	OH
11**HEI2**	OH	H	CH_3_	H	I	CH_3_	I	OH
11**HEI3**	OH	I	CH_3_	H	I	CH_3_	I	OH
12	H	CHO	OH	H	H	H	H	H
13	OH	H	H	H	OH	H	H	H
14	OH	H	CH_3_	H	H	OCH_3_	H	OH
15	OH	OH	H	H	H	H	H	H
16	OCH_3_	OH	H	H	H	H	H	H
17	OH	H	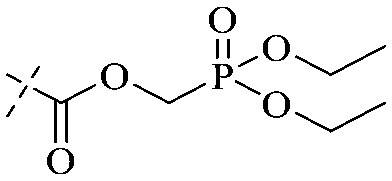	H	H	H	H	OH
18	OH	H	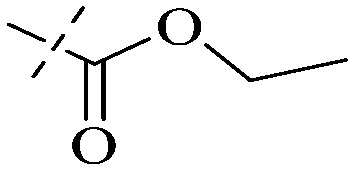	H	H	H	H	OH
19	OCH_3_	H	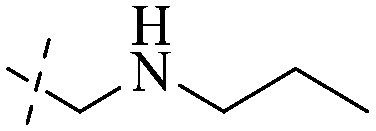	H	H	H	H	OH
20	OH	H	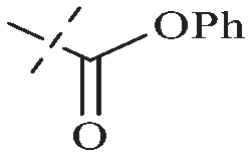	H	H	H	H	OH
21	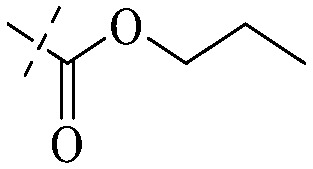	H	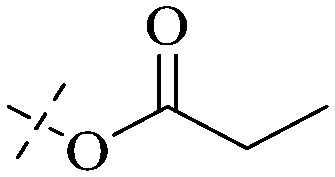	H	H	H	H	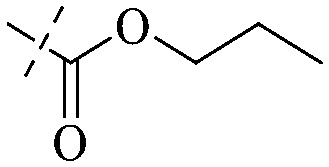
22	OCH_3_	H	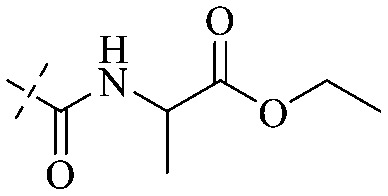	H	H	H	H	OCH_3_
23	OCH_3_	H	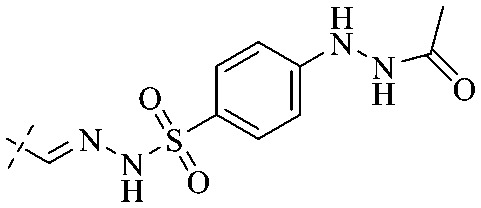	H	H	H	H	OCH_3_
24	OCH_3_	H	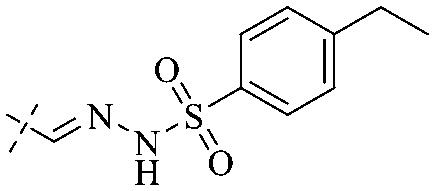	H	H	H	H	OCH_3_
25	H	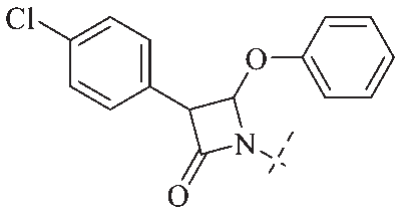	H	H	H	H	H	H
26	OH	CH_3_	OH	H	H	OH	H	H
27	OH	CH_3_	OH	H	H	OH	H	H
28	OH	CH_3_	H	H	H	OH	H	H
29	OH	OH	H	H	H	H	H	H
30	OH	CH_3_	OH	H	H	H	H	H
31	OCH_3_	CH_3_	OH	H	H	H	H	H
32	OH	CH_3_	OCH_3_	H	H	H	H	H
33	OH	CH_3_	H	H	H	H	H	H
34	OH	CHO	H	H	H	H	H	H
35	OH	CHO	OH	H	H	H	H	H
36	OCH_3_	CHO	OH	H	H	H	H	H
37	OH	CH_2_OCH_3_	OH	H	H	H	H	H
38	OCH_3_	CH_3_	OCH_3_	H	H	H	H	H
39	OH	H	OCH_3_	H	H	H	H	H
40	OCH_3_	H	OCH_3_	H	H	H	H	H
41	OH	OH	H	OH	H	H	H	H
42	OH	CH_2_CH_3_	H	H	H	H	H	H
43	OH	H	OH	H	H	H	H	H
44	H	CH_3_	H	H	H	H	OH	H
45	OH	CH_2_OH	OH	H	H	H	H	H
46	OH	CH_2_OC_2_H_5_	OH	H	H	H	H	H
47	OH	CH_2_OC_2_H_5_	H	H	H	H	H	H

**Table 2:** *In vitro* antibacterial activity of emodin (μg mL^−1^)

Strain (object)		MIC (μg mL^−1^)	Ref.
Gram-positive bacteria	*Staphylococcus aureus*	64	34
	*Staphylococcus aureus* CMCC26003	0.125	44
	*Bacillus* species	0.5–2.0	35
	Methicillin-resistant *Staphylococcus aureus*	4	36 and 39
	*Bacillus subtilis*	8–32	37 and 38
	*Mycobacterium tuberculosis*	0.9	40
	*Bacillus cereus* TISTR 687	16	41
	*Streptococcus suis* strain ATCC700794	0.125	43
Gram-negative bacteria	*Escherichia coli*	>128	34
	*Pseudomonas aeruginosa*	>128	34
	*Pseudomonas aeruginosa* TISTR 781	128	41
	*Salmonella typhimurium* TISTR780	128	41
	*Haemophilus parasuis*	32	42

Chrysophanol (2) is involved in a variety of biological activities, including anti-bacterial, anti-cancer, anti-virus, anti-diabetes, anti-inflammatory, anti-ulcer, anti-obesity and liver protection.^45,46^ The antileishmanial activity of chrysophanol against chloroquine-resistant (IC_50_ = 20.13 μg mL^−1^) and chloroquine-sensitive strains of *P. falciparum* (IC_50_ = 7.80 μg ml^−1^) was exhibited.^47,48^ Coopoosamy also observed an inhibitory effect of chlorophenol on intestinal infection in mice (IC_50_ = 12.70 μg ml^−1^).^49^ Its antibacterial effects proposed by recent studies may be achieved by destroying biofilms.^50,51^ The antibacterial activity of chrysophanol is demonstrated in Table 3.

**Table 3:** The *in vitro* antibacterial activity of chrysophanol (μg mL^−1^)

Strain (object)		MIC (μg mL^−1^)	Ref.
Gram-positive bacteria	*Bacillus cereus*	>250	52
	*Staphylococcus aureus*	>250	52
	*Staphylococcus epidermidis*	31.25	52
	*Mycobacterium tuberculosis* H37Ra	64	60
	*Mycobacterium tuberculosis bovis*	64	60
Gram-negative bacteria	*Escherichia coli*	125	52
	*Escherichia coli*	3.13	58
	*Aeromonas hydrophila* IB101	200	53
	*Aeromonas hydrophila*	200	53
	*Micrococcus kristinae*	>250	54
	*Proteus vulgaris*	128	54
	*Enterobacter aerogenes*	>250	54
	*Pseudomonas aeruginosa*	128	55
	*Vibrio harveyi*	1000	56
	*Neisseria gonorrhoeae*	75	59
Fungus	*Trichophyton rubrum*	156	52
	*Epidermophyton floccosum*	625	52
	*Candida albicans*	50	57
	*Cryptococcus neoformans*	50	57

Aloe emodin (3) displays extensive pharmacological effects. Its antibacterial effect was accomplished by acting on the initial adhesion and proliferation of biofilm development.^61,62^ When bacterial cells were treated with aloe emodin, the changes of the genes related to cell thiometabolism, lysine and peptidoglycan biosynthesis and biofilm formation took place. The decrease of *N*-acetyltransferase (NAT) activity in the cytoplasm of *Helicobacter pylori* was dose-dependently associated with the increase of aloe emodin (Table 4).^63^ A carbon nanoparticle polymer hybrid hydrogel loaded with an aloe emodin^64^ quickly generated a large amount of heat and active oxygen with the help of near-infrared radiation, achieving controllable bacteriostasis. The latest research^65,66^ unfolded that aloe emodin-containing waterborne polyurethane was a good antibacterial agent.

**Table 4:** The *in vitro* antibacterial activity of aloe emodin (μg mL^−1^)

Strain (object)		MIC (μg mL^−1^)	MBC (μg mL^−1^)	Ref.
Gram-positive bacteria	*Staphylococcus aureus* species	16–32	64–128	62
	*Staphylococcus epidermidis* BNCC102555	32	128	
	*Staphylococcus epidermidis* ATCC12228	4	16	
	*Streptococcus pneumoniae*	16	64	

Rhein (4) exhibits good antibacterial activity.^67^ It was first reported^68^ that rhein can inhibit NAT activities of the bacteria *Helicobacter pylori*. At the concentration of 1.5–25 mg mL^−1^, 4 was able to inhibit *staphylococcus*, *streptococcus*,^69^*diphtheria*, *Bacillus subtilis*, *paratyphoid bacillus*, *dysentery bacillus*, *etc.* Its mechanisms included the inhibition of electron transfer in the mitochondrial respiratory chain, a strong inhibitory effect on nucleic acid and protein synthesis. Also, 4 was found to have a very good affinity for bacterial DNA/CpG DNA and was capable of inhibiting LPS-induced TNF-α release from RAW264.7 cells in a dose-dependent manner.^70^ When treated with rhein,^71,72^ the bacterial morphology was influenced, the integrity of the cell wall was disrupted, biofilm formation was blocked, and bacterial metabolism was decreased. Additionally, the bacterial glycolysis pathway was significantly affected by rhein,^73,74^ which was associated with an effect on the activity of type II NADH: quinone oxidoreductase (NDH-2).^75,76^ The corresponding antibacterial activities are summarized in Table 5.

**Table 5:** The *in vitro* antibacterial activity of rhein (μg mL^−1^)

Strain (object)		MIC (μg mL^−1^)	Ref.
Gram-positive bacteria	*Staphylococcus aureus*	4–16	69
	*Streptococcus mutans*	6.25	77
Gram-negative bacteria	*Escherichia coli*	125	78
	*Salmonella*	250	78
	*Porphyromonas gingivalis*	2.5	79

The antibacterial effects of emodin, rhein and aloe emodin are generally more potent than those of emodin methyl ether and chrysophanol. Structurally, these anthraquinones having the same parent nucleus are different from their substituents on C-3 and C-6. The polar substituents including –COOH, –OH and –CH_2_OH attached to rhein, emodin and aloe emodin are beneficial to the improved antibacterial activity, while the electron-donating groups, –OCH_3_ and –CH_3_, introduced to emodin methyl ether and chrysophanol may weaken the antibacterial activity.^81^ By comparing several anthraquinones against *B. adolescentis*,^82^ the stronger the polarity of the substituent is, the better the antibacterial effects occur. A similar result is postulated by the comparison of the antibacterial activities of rhein and aloe emodin.^83^ 1,8-Dihydroxyl groups contribute to the antibacterial activity of anthraquinone derivatives due to the generation of free electrons.^80^ The antibacterial activity of more anthraquinone derivatives from the root extract of *Huangmu Bacopa monniera* demonstrated a hydroxyl group introduced on C-8 has a catalytic effect, and its removal led to a decrease in the antibacterial activity.^85^ However, the introduction of electron-donating groups to 1,8-dihydroxyoxanthraquinone resulted in decreased antibacterial activity, especially against MRSA.^84^ Interestingly, in Lee's study, two electron-donating group hydroxyl groups of 1,2-dihydroxyanthraquinone were shown to be essential in breaking the bacteria membrane,^86^ emphasizing an important role of the two hydroxyl groups at positions C-1 and C-2 of anthraquinone played in the antibacterial activity. The long aliphatic chain substituted on C-6 of rhodopsin enhanced its antibacterial properties, while the substitution residing in its C-2 lowered the antibacterial activity.^87^ The introduction of various long chains to the anthraquinone structure of emodin facilitated the compounds to disrupt the bacterial membrane and increase the antibacterial activity, probably ascribed to the enhanced lipophilicity making it easier to bind the biofilm.^88^ Although the presence of the long aliphatic chain in the emodin structure increased the antibacterial activity, the methoxy group introduced was not conducive to the bactericidal activity. In terms of the decreasing antimicrobial activity, the rank is 5 > 7 > 8 > 6.

In addition to the polarizability, the antimicrobial properties of anthraquinone derivatives rely on the pH of the environment and the number of hydrogen bond acceptors.^89–92^ Basu and Duan's research studies^93,94^ revealed that the antibacterial activity of 9 was generally better than that of 1, but 1 was inferior to 10. Derivative 11 where the emodin was replaced by iodine displayed improved antibacterial activity against MRSA and other strains, and its increased ability to destroy the bacterial membrane.^95^ The antibacterial activity of compound 12 having formaldehyde and citrin introduced in the structure of emodin was not reported.^96^ Comparing compounds 1–4 and 13 against the *T. vaginalis* G3 strain, the introduction of phenolic hydroxyls at 1,2,4 positions of the benzene ring on the same side of anthraquinone led to reduced activity.^97^ Compounds 1, 2 and 14, isolated from *Senna macranthera* roots, exhibited potential antibacterial activities against *Staphylococcus aureus* strains from animals suffering from mastitis infections with MIC values of 20, 90, and 90 μg mL^−1^,^98^ respectively. The design and synthesis of 20 aloe emodin derivatives similar to the structure of 15 were demonstrated in general, and the activity of four compounds was tested, suggesting improved activity ascribed to the electron donor group at positions 1 and 2 of anthraquinone.^99^16 with novel structural aloe-emodin azoles as a potential antibacterial agent exhibited lower toxicity and higher antibacterial activity.^100^ A series of emodin derivatives similar to the structure of 17 with anti-MRSA activity were designed and evaluated.^101^ Additionally, the target compounds 18–22 showed different levels of antifungal activity.^102^ Noticeably, some showed higher inhibitory activity against *R. solani*, in comparison with the parent compound rhein. From their preliminary structure–activity relationships, the antifungal activity of rhein amide was higher than that of rhein ester. Replacement of hydroxyl groups at positions 1 and 8 resulted in a decrease in the antibacterial activity. The hydroxyl group at the R_1_ position was important and necessary for the activity. Aloe emodin conjugated 23 and 24 with sulfonylhydrazone as new antibacterial regulators also highlighted that the introduction of electron-donating substituents at R_2_ and R_3_ positions could improve the activity and reduce hemolytic toxicity.^103^ A series of compounds similar to derivative 25 have been synthesized for antibacterial evaluation, and as a result, the existence of the methylthio substituent at C-3 and the 3,4,5-trimethoxyphenyl group at C-4 of the β-lactam ring significantly increased the antibacterial activity.^104^ The antibacterial activity of compounds 26–28 and purpurin (41) against *S. aureus* and *C. albicans* was evaluated, indicating that 28 and 41 are potential drugs for photodynamic antibacterial chemotherapy.^105,106^

##### 2.1.1.2 Alizarin type anthraquinones

This type of anthraquinone has hydroxyl groups distributed on one side of the benzene ring, and includes alizarin, hydroxyalizarin and pseudo hydroxyl alizarin in the traditional Chinese medicine *Rubia cordifolia*. Alizarin type anthraquinones, the main pharmacodynamic component of *Rubia cordifolia* and *Morinda officinalis*, have certain clinical medicinal value and wide application.^107,108^*Rubia* anthraquinones primarily originate from *Rubia cordifolia Linn* (*Rubiaceae*), including compounds 29–32.^109^ The antibacterial activity of compounds 33–40 was tested, displaying that 34, 35 and 40 had certain antibacterial ability.^110^41, one of the two chemical markers, which serves to evaluate the quality of herbal medicines in the Chinese Pharmacopoeia,^111^ inhibited the growth of Gram-negative and Gram-positive bacteria, Ape with IC_50_ values ranging from 0.3 to 23 μM.^112^ Some anthraquinones 42–47 had mutagenic activity against *Salmonella typhimurium*.^113^ From the above analysis, the structure–activity relationships of emodin and alizarin and their derivatives are summarized in Fig. 2.

#### 2.1.2 Anthracenol, anthrone and their derivatives

**Fig. 3:**
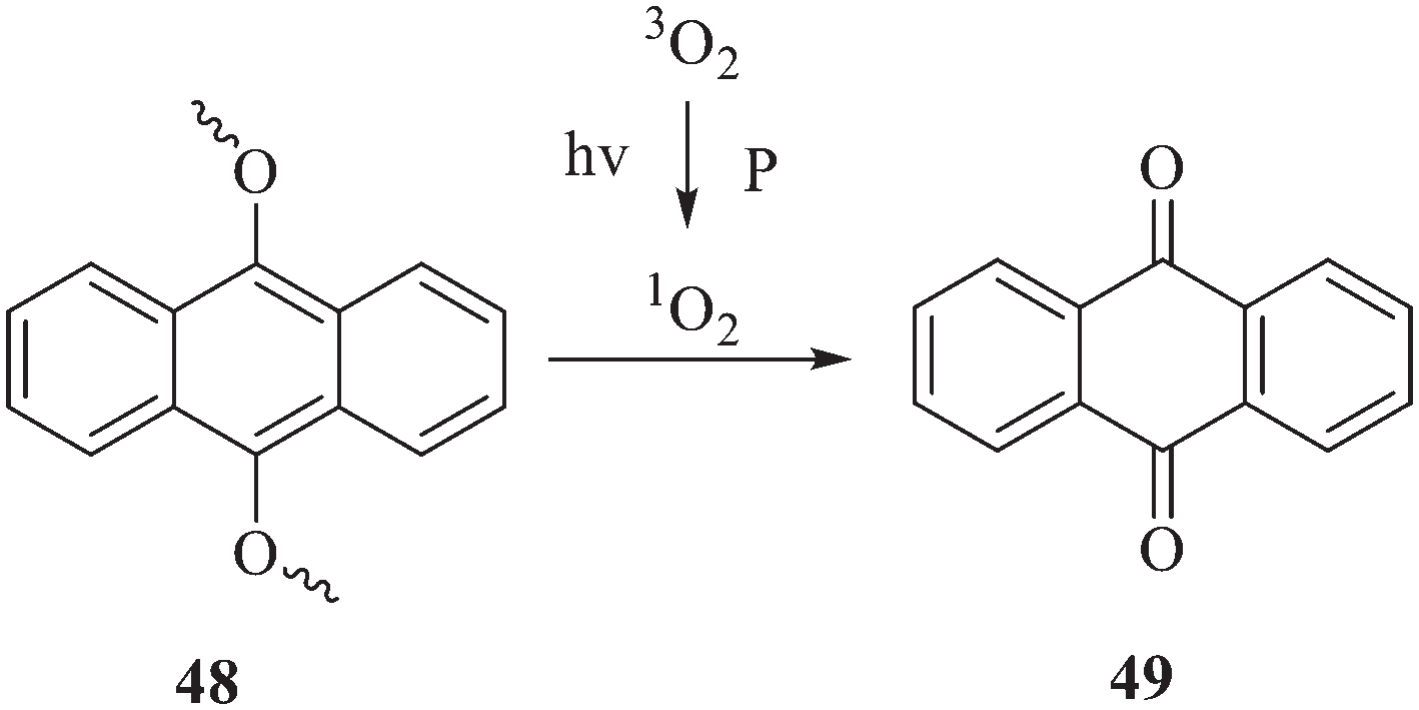
Cleavage mechanism of 9,10 monoalkoxy anthracene under the action of singlet oxygen.

Anthraquinone can be reduced by zinc powder in alkaline solution to produce reduced anthraquinone and its tautomer anthraquinone. Both reduced anthraquinone and anthraquinone are unstable, the reduced anthraquinones are easily oxidized to anthrone or anthraquinone, and anthrone is easily oxidized to anthraquinone, so the reduced anthraquinones are rarely found in plants. Fresh rhubarb contains anthracene phenols, which are undetectable when stored for more than 2 years. In acidic solution, the reduced anthraquinone and its tautomer anthrone are formed. The anthraquinone derivative is relatively stable when the hydroxyl group at the *meso* position is condensed with a sugar to form a glycoside. Only removal of a glycosyl group easily leads to oxidation into anthraquinone, hinting an electron transfer occurring between anthracene phenol and anthrone. As shown in Fig. 3, a dissociating monoclinic oxygen-sensitive linker 9,10-dialkoxyanthracene that contains hydrogen or other carbon substituents on 9,10 sites of anthracene is an efficient, reliable, and rapid functional site for capturing singlet oxygen.^114–116^ Based on this feature, it can be developed into fluorescent dyes or antibacterial agents that inhibit respiratory electron transfer chains. To date, only a few reports have focused on the antibacterial activity of anthracene phenols, anthracene and its derivatives 50–52, for instance, xanthone derivative 50 was demonstrated to display a variety of biological activities and medical value.

The synthesis of 12 alkyl amino substituted azabenzoanthrone derivatives, like 53, was reported by the Tang research group.^117^ The antibacterial evaluation unfolded that compounds 53Ia–Ij exhibited strong inhibitory activity against *S. aureus*, *B. megaterium*, *S. typhimurium* and *B. subtilis*, and the activities of 53Ia and 53Ib were more potent. Compounds 54–76 are listed in Table 6. The antibacterial activity of anthrone derivatives 54–62 is discovered to be related to the number of substituents on the two aromatic rings. The hydroxyl groups of rings A and C are very important for the antibacterial activity, and alkylation of the hydroxyl groups of C-3 and C-6 decreases the antibacterial activity. The longer the alkyl chain is, the more the antibacterial activity decreases.^118^ Similarly, a series of alkene xanthones 63–71 extracted from *Garcinia staudtii Engl.* are tested against methicillin-resistant *S. aureus*, exhibiting strong antibacterial and immunomodulatory abilities.^119^

**Table 6:** Structures of anthraquinone derivatives

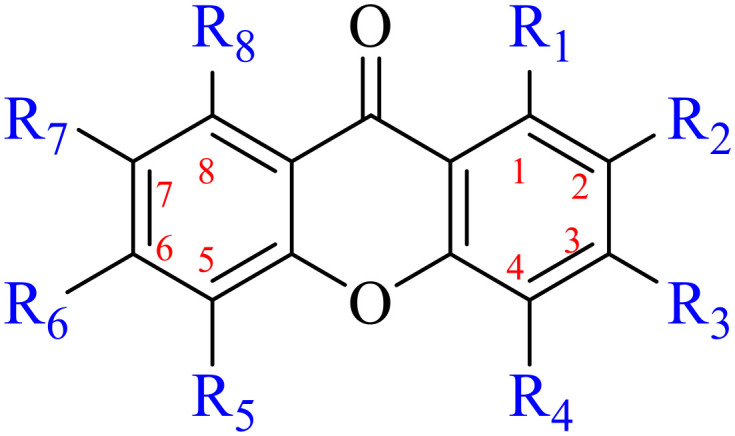								
Name	R_1_	R_2_	R_3_	R_4_	R_5_	R_6_	R_7_	R_8_
54	H	H	H	H	H	H	OH	OCH_3_
55	H	H	H	H	H	H	OCH_3_	OCH_3_
56	H	H	H	H	OCH_3_	OH	H	H
57	H	H	H	H	OCH_3_	OCH_3_	OCH_3_	OCH_3_
58	OH	H	H	H	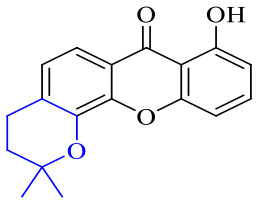		H	H
59	OH	OCH_3_	OH	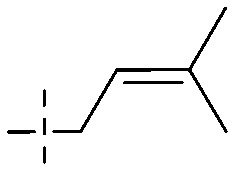	OH	H	H	H
60	OH	H	H	H	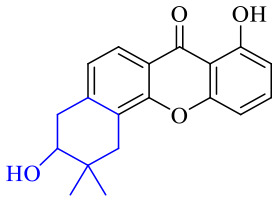		H	H
61	OH	H	H	H	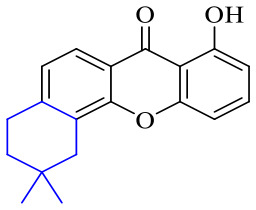		H	H
62	OH	H	H	H	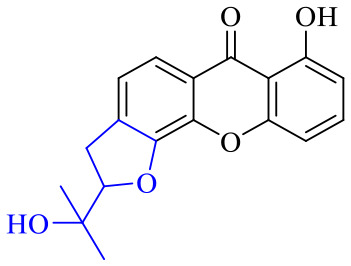		H	H
63	OH	Prenyl	OH	Prenyl	OH	OH	OH	OH
64	OH	Prenyl	OH	H	H	OH	Prenyl	OCH_3_
65	OH	Prenyl	OH	Prenyl	OH	H	H	OH
66	OH	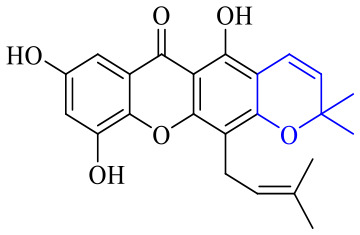		Prenyl	OH	H	OH	H
67	OH	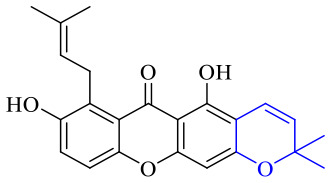		H	H	H	OH	Prenyl
68	OH	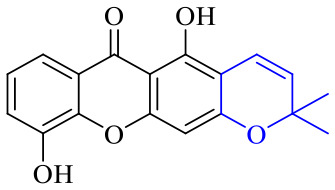		H	OH	OH	H	H
69	OH	H	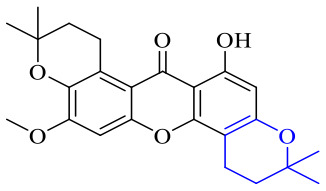		H	OCH_3_	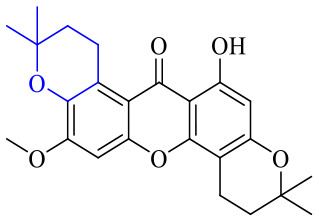	
70	OH	Prenyl	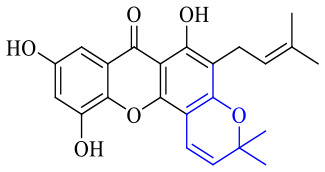		OH	H	OH	H
71	OH	Prenyl	OH	H	H	OH	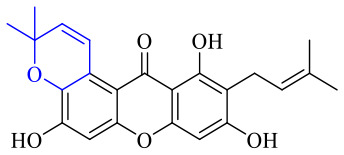	
72	H	H	H	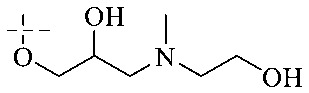	H	H	H	H
73	H	H	H	CH_3_	H	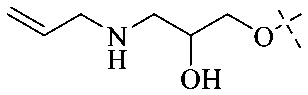	H	H
74	H	H	H	CH_3_	H	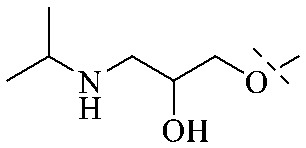	H	H
75	H	H	H	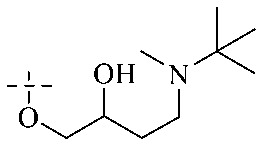	H	Cl	H	H
76	H	H	H	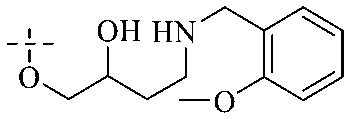	H	H	H	H

**Fig. 4:**
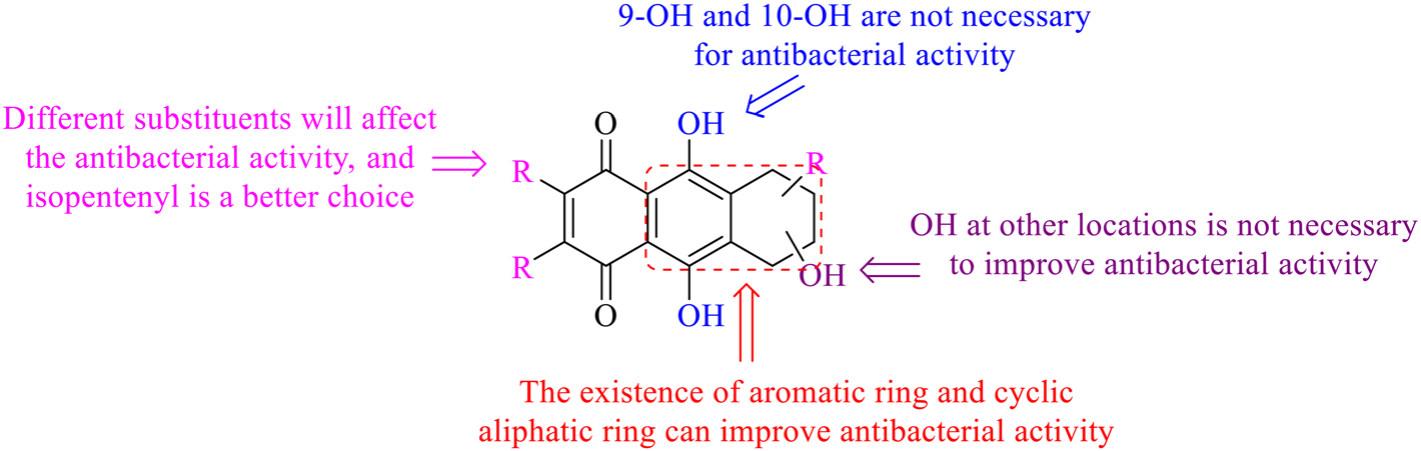
Antibacterial structure–activity relationship of hydrogenated anthraquinone derivatives.

Of anthrone derivatives 72–76 synthesized, the lowest MIC was below 20 mg L^−1^.^120^ The structure–activity relationship analysis showed that the existence of two hydroxyl groups in the amine part was necessary in the anti-*Helicobacter pylori* activity, and the activity depended more on the structure and configuration than on the hydrophilic properties. Compounds containing a tertiary butylamine substructure displayed higher activity than the ones with an isopropylamine fragment. The structure–activity relationship of oxanthrone derivatives was not discussed here, but refer to ref. 149. The antibacterial activity of 77 and 78 was tested and found to present moderate antibacterial activity against bacteria such as *B. subtilis*, *Bacillus cereus*, *S. aureus*, *Escherichia coli*, *P. aeruginosa* and *S. sonnei*.^120,121^ The *in vitro* antibacterial activity of compounds 79–86 was evaluated, and as a result, 83 had obvious antibacterial activity against *B. subtilis* with a MIC value of 312 nM, and the activity of 85 against *Bacillus cereus* was more potent (MIC = 8.8 nM).^122^ Therefore, the antibacterial SAR of hydrogenated anthraquinone derivatives is summarized in Fig. 4.
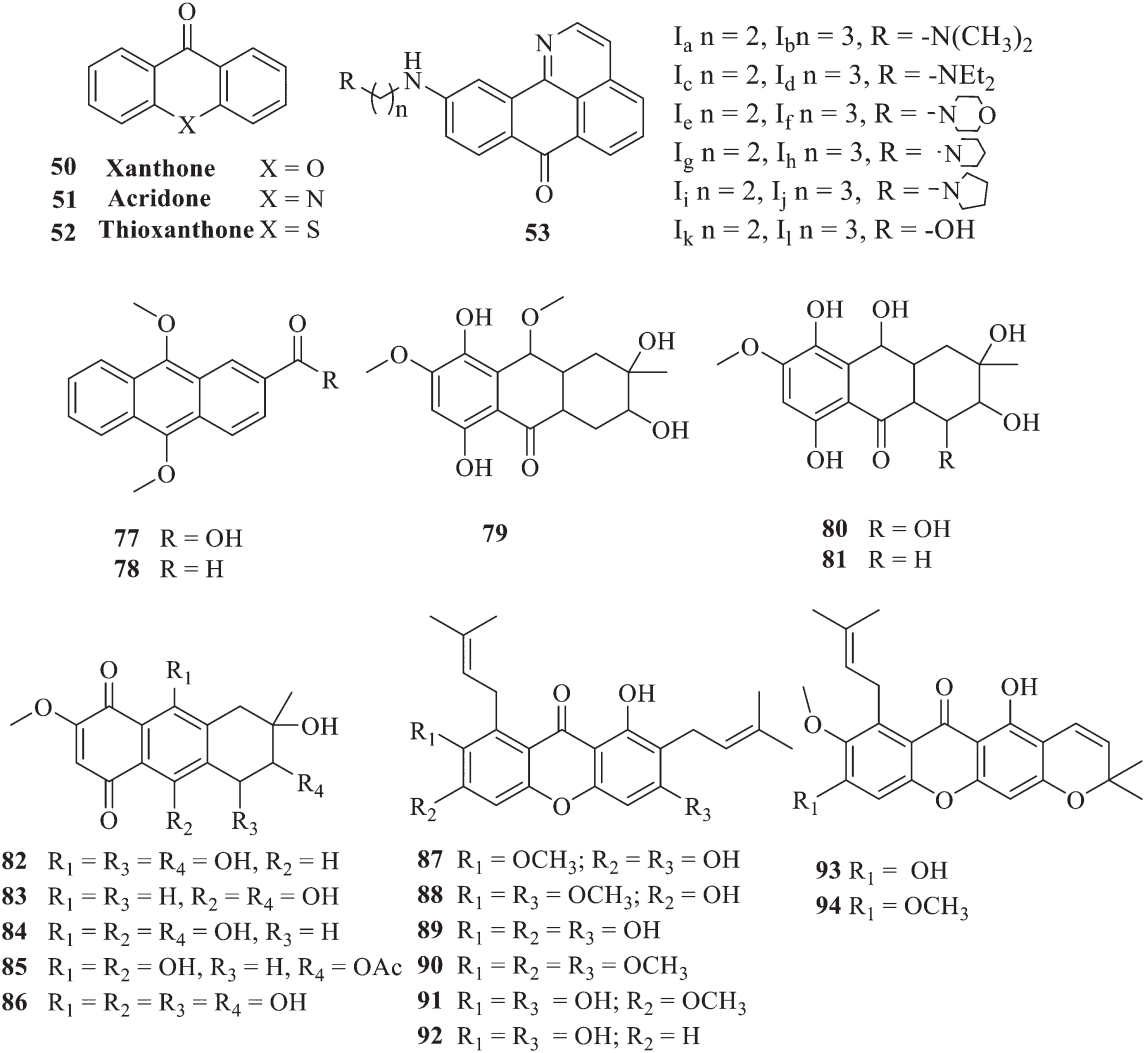


**Fig. 5:**
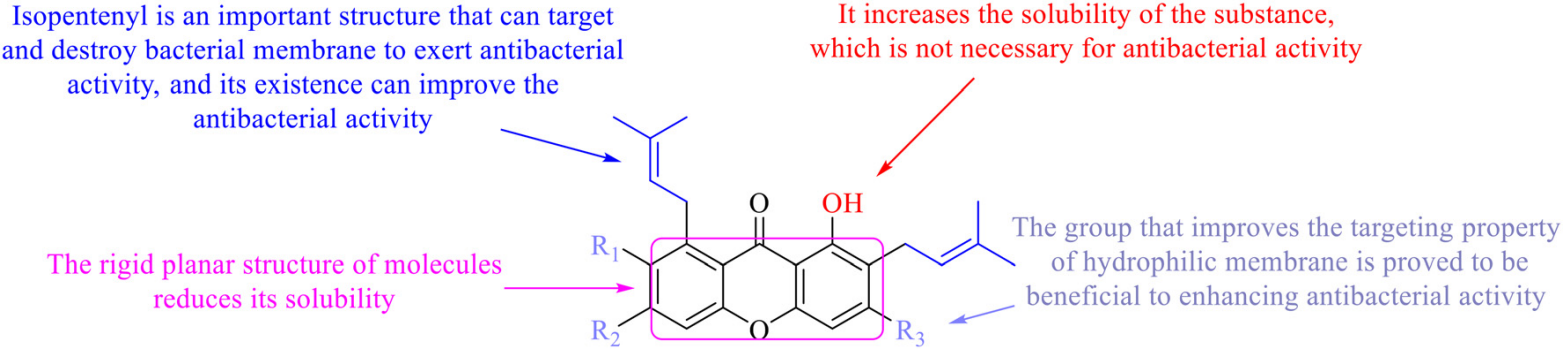
Antibacterial structure–activity relationship of mangostin derivatives.

Next, mangostin and its derivatives^123^87–94 have been explored. The MICs of mangostin (88) against MRSA, MSSA, VRE and VSE are 3.13, 6.25, 6.25 and 6.25 mg mL^−1^, respectively, along with little toxicity and few side effects. Mangostin having two di-isopentenyl scaffolds and one xanthone core exerts excellent antibacterial activity *via* a membrane targeting manner, implying mangostin is a promising lead for developing antibacterial candidates. Its SAR is shown in Fig. 5.^124,125^

**Fig. 6:**
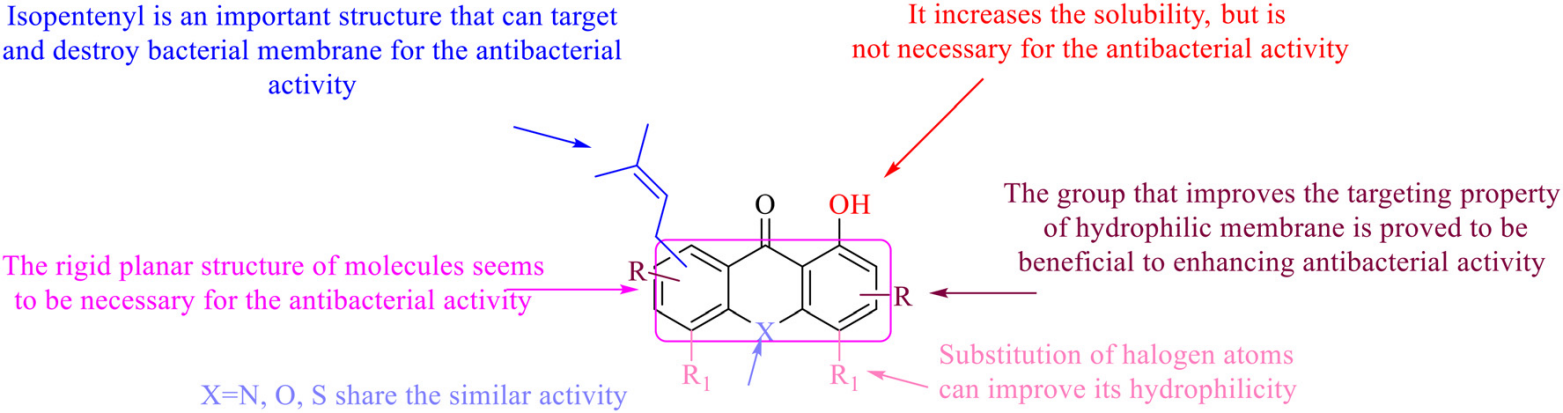
Structure–activity relationship of oxaanthone, thiaanthone, azaanthone.

Compound 95 showed inhibitory activity against *S. aureus* with the diameter of its bacteriostasis ring equal to 9.58 mm.^126^ Some ketinone derivatives 96–104 (ref. 127) were prepared, and 99–101 exhibited broad-spectrum antifungal effects. Through the analysis of the structure–activity relationship, the presence of the linear amine at C-1 of the thioxanthone scaffold seemingly was the pharmacological feature, while the nature of the substituent at C-4 failed to inhibit fungal growth. More than 40 anthrone derivatives were screened, and 96, 105 and 106 demonstrated antibacterial activities against a MRSA isolate with MIC values of 32–256 mg ml^−1^.^128,129^ The SARs of thioxanthone, acridone and their derivatives are summarized in Fig. 6.
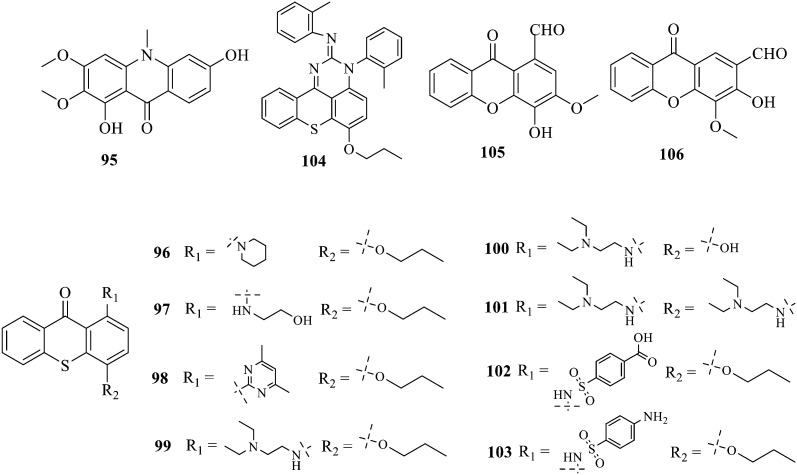


Recently, cationic anthraquinone analogs (CAAs) have been demonstrated to hold great and excellent potential for antibacterial activity. CAAs 107–113 mainly exerted their antibacterial activity by disrupting the redox process. At high concentrations, these compounds also served as membrane breaking agents.^130^ The functional groups on N-1 played a crucial role in regulating the biological characteristics and the biological activity of these molecules. Of note, CAAs containing linear alkyl groups had good antibacterial activity, while CAAs carrying aromatic groups exhibited good anticancer activity. Additionally, when the N-1 and N-3 positions of CAAs were replaced by alkyl chains with various lengths, the structural characteristics of naphthoquinones including the core nucleus, cations, and oxygen-containing alkyl chains probably affected the antibacterial activity. Moreover, the antibacterial activity against Gram-positive bacteria was by far higher than that against the Gram-negative ones.^131,132^ Collectively, CAAs exhibited an adjustable activity and selectivity, opening the way to develop broad-spectrum antibiotics.

We have acquired the whole genome of the Gram-negative bacteria, but the finding does not create new antimicrobial agents. Anthraquinone derivatives serving as antibacterial agents do not benefit from the whole genome. Moderate antibacterial activities of compounds 77 and 78 towards selected Gram-positive and Gram-negative bacteria were observed. 2 can also inhibit *Pseudomonas aeruginosa* and *Escherichia coli*, and its growth inhibition zone was 18 mm.^42^ The MIC of aloe emodin against *E. coli* and *P. aeruginosa* ATCC 27853 was 128–259 μg ml^−1^.^62^ Interestingly, the antibacterial mechanism of anthraquinone derivatives against Gram negative or positive bacteria was demonstrated to be similar.
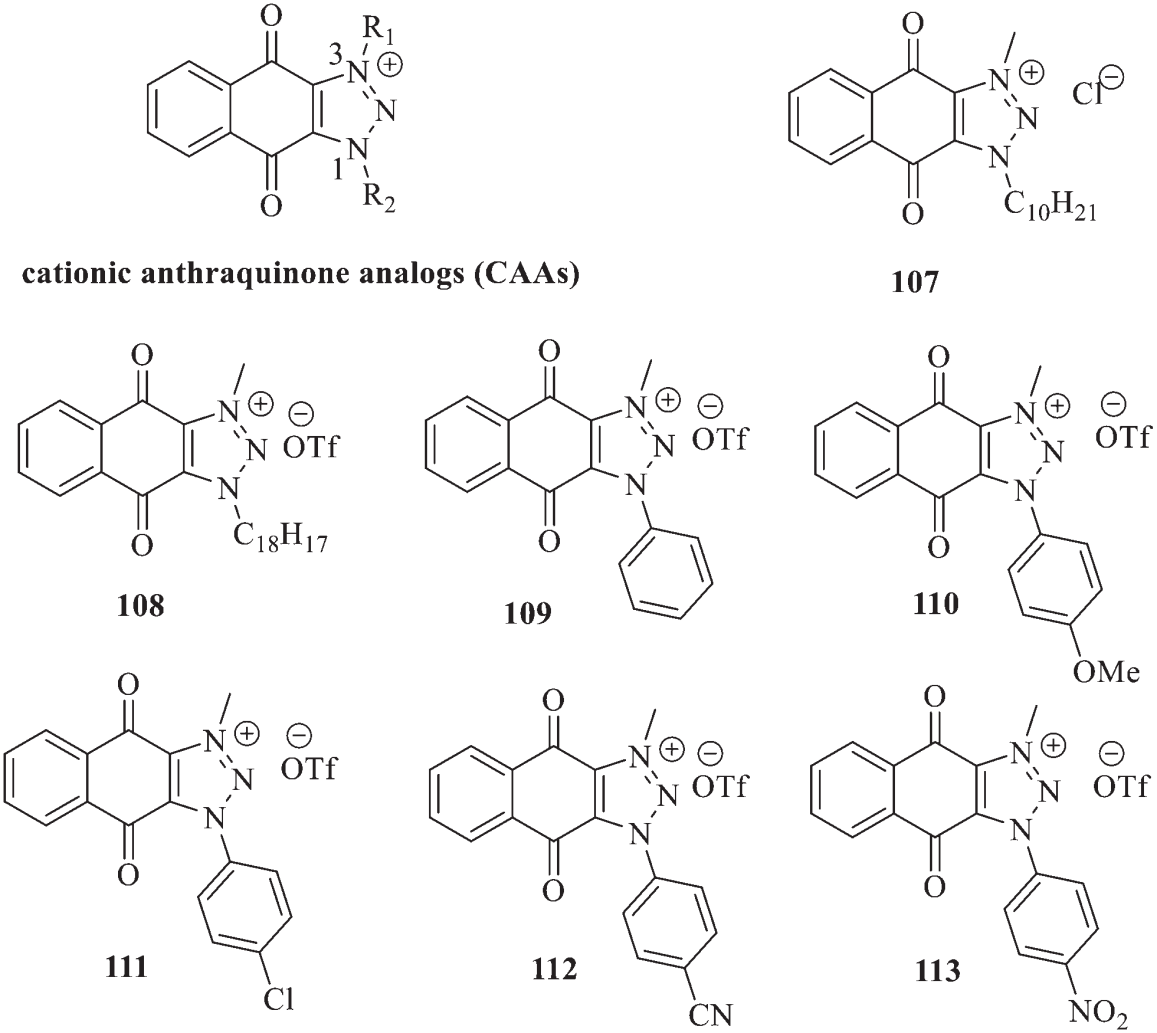


### 2.2 Binuclear anthraquinone

Two single anthraquinones are dehydrated and condensed to form double anthraquinones in two approaches. The structure of anthraquinones is different according to the different dehydration water and dehydration positions. One way is to react between positions C-4, C-5 and C-10 to remove two dihydroxides at the same time; the other way is that the dehydration condensation reaction between positions C-5, C-6 and C-7 of two molecules and removal of one water molecule form hypericin derivatives 114–123.^133^ Hypericin 114 is a widely-studied natural double anthraquinone from *Hypericum japonicum*. It is involved in strong biological activities such as antiviral, antidepressant and photodynamic activities.^134^ Unfortunately, only the cytotoxicity of 114–123 was reported and no data on the antibacterial activity^135,136^ were referred to. The xanthone dimer derivative garmoxanthone 124 showed strong inhibitory activity against MRSA ATCC43300 (MIC = 3.9 μg mL^−1^) and MRSA CGMCC1.12409 (ref. 137) (MIC = 3.9 μg mL^−1^), and exhibited moderate activity to the tested *vibrio* strain with MICs ranging from 15.6 to 31.2 μg mL^−1^. 125 and 126 with a rare C–O–C ether bond dimerization demonstrated selective antibacterial activity against Gram-positive *Staphylococcus aureus*.^138^ The structure–activity relationship of anthraquinone derivatives including 127 revealed that a long fatty chain and a methoxy group contained in the substituent could improve the antibacterial activity.^139^ It is noted that the SAR of binuclear anthraquinone is similar to that of mononuclear anthraquinone in the antibacterial activity.
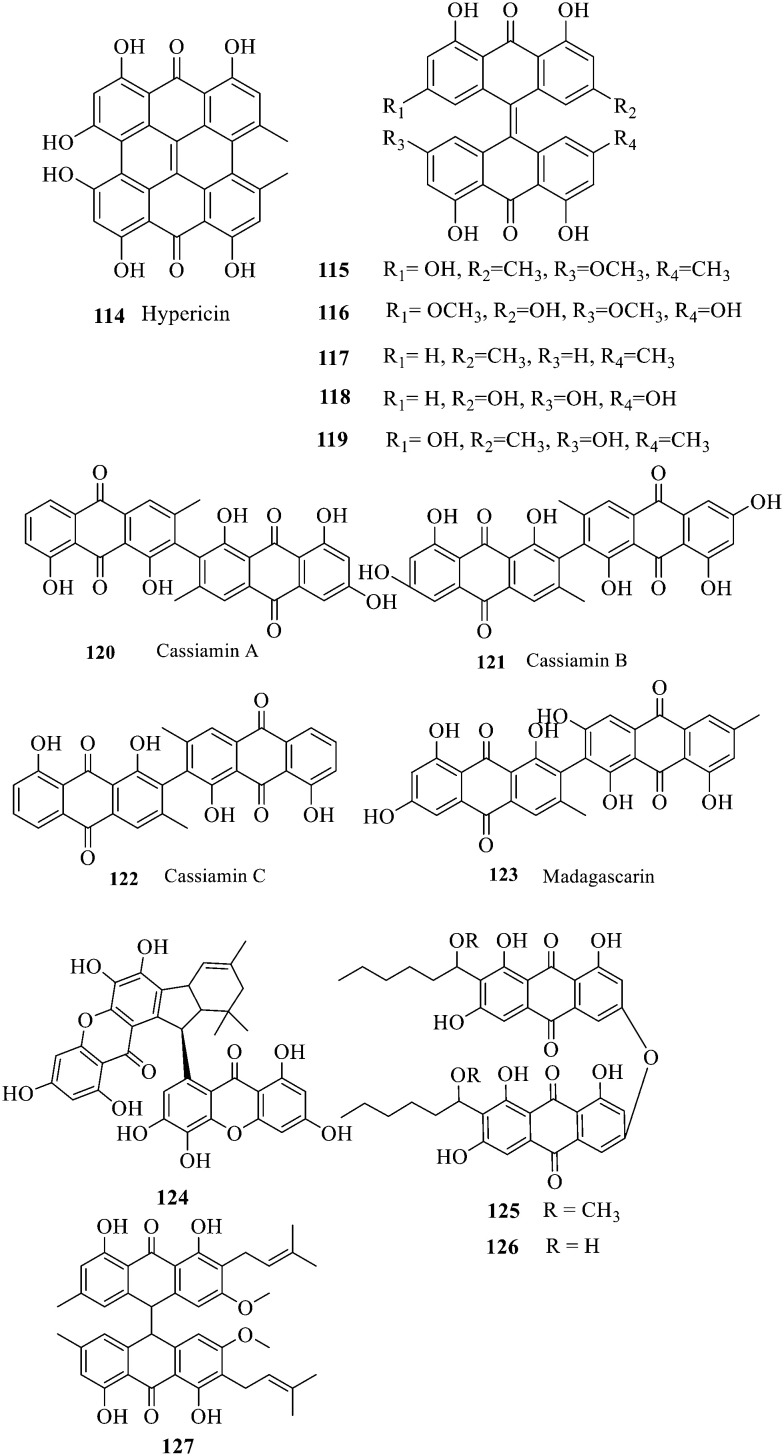


### 2.3 Anthraquinone glycosides

#### 2.3.1 Aloin-based glycosides

Aloin, also known as *Aloe vera*, is a natural organic compound. Extensive attention has turned to its anti-inflammatory, anti-cancer, antibacterial and antioxidant activities. Sennosides 128–130, aloin 131 and mangiferin 132 are common anthraquinone glycosides, holding great antibacterial potential.^140,141^ For details of anthrone glycosylation, refer to ref. 165. The antibacterial activity of 131 and 133 against 23 kinds of bacteria and four kinds of fungi was tested. As a consequence, 133 showed a certain activity against multi-drug resistant *Staphylococcus aureus* (NCTC 11994) and *Salmonella typhimurium* (ATCC 1255) with MIC values of 0.72 and 0.18 mM, respectively.^142^ However, the antibacterial effect of 134 and 135 was ineffective.^143^ Generally speaking, the introduction of a glycosyl group improved the water solubility and the activity of anthraquinone, implying glycosylation as an effective method in the antibacterial activity. Similarly, the structure–activity relationship of anthraquinone glycosides followed that of mononuclear anthraquinone. The antibacterial mechanism of aloin as a tetracycline analogue was similar to that of aminoglycosides, inhibiting bacterial protein synthesis by blocking ribosome response sites.^144^ Aloin can inhibit *C. neoformans* and display synergistic antibacterial activity when co-administered with amphotericin B.^145^ Also, the activity of anthraquinone glycosides against *mycobacterium*^146^ was observed. Recently, aloe has been reported to show a significant inhibition effect on plaque formation of *Porphyromonas gingivalis* and *Actinobacillus actinomycetes* in 30 patients suffering from periodontitis.^147^ The detailed antibacterial activity of anthraquinone glycosides is shown in Table 7.
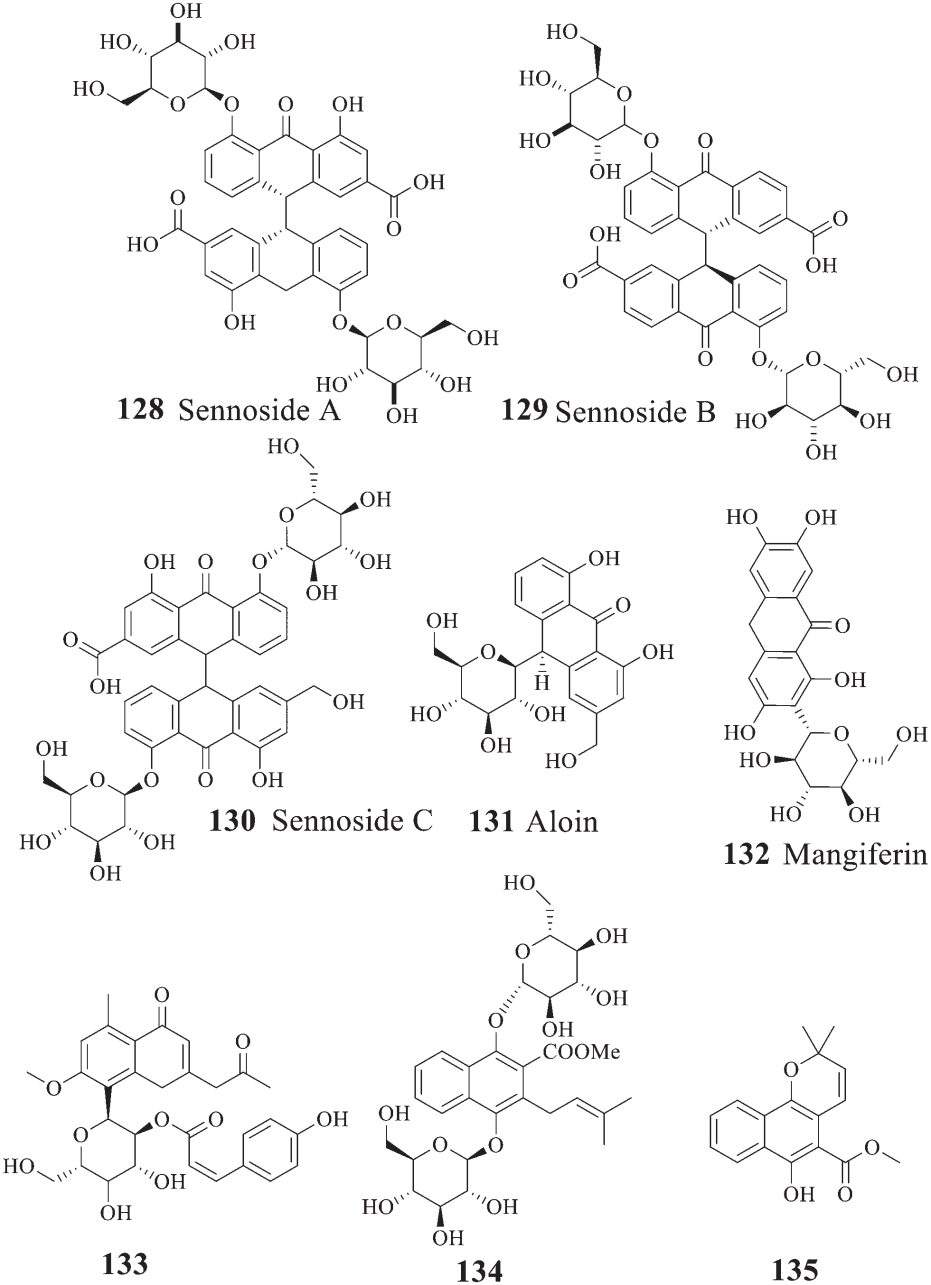


**Table 7:** The *in vitro* antibacterial activity of aloin and its glycosides (μg mL^−1^)


Strain (object)		MIC (μg mL^−1^)	Ref.
Gram-positive bacteria	*Bacillus subtilis*	240	148
	*Streptococcus*	120	148
	*Staphylococcus aureus* ML267	1910	148
	*Mycobacterium tuberculosis* H37Ra	32	146
	*Streptococcus sobrinus*	2.5	149
Gram-negative bacteria	*Bacillus pumilus*	120	148
	*Escherichia coli*	60–120	148
	*Salmonella typhi* Ty2	60	148
	*Shigella boydii* D13629	960	148
	*Vibrio cholerae*	120	148
	*Mycobacterium bovis*	128	146
Fungus	*Cryptococcus neoformans*	64	145

## 3. Antibacterial mechanisms and toxicity of anthraquinones

### 3.1 Antibacterial mechanisms of anthraquinones

#### 3.1.1 Intervention or destruction of biofilms

**Fig. 7:**
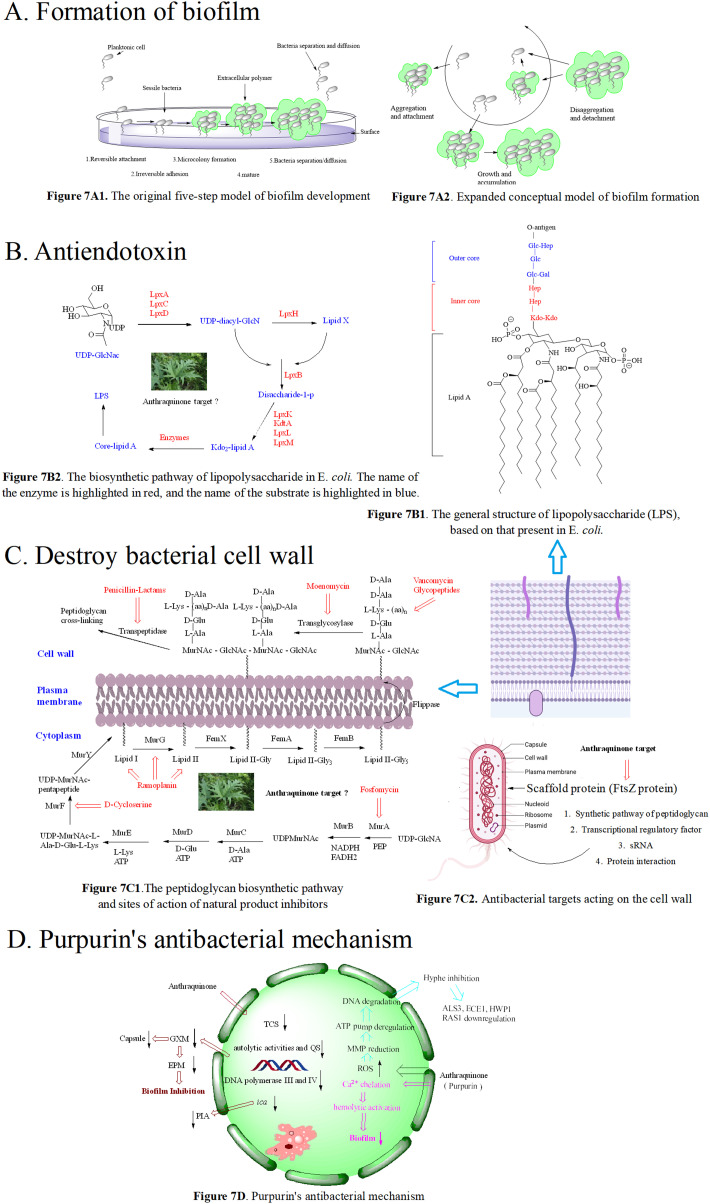
7A1 The original five-step model of biofilm development. 1) In the reversible attachment stage, bacteria attach to the substrate surface non-specifically; 2) in the irreversible attachment stage, bacteria interact with the substrate surface through adhesion protein or adhesion factor; 3) at the micro colony formation stage, bacteria produce extracellular polymers; 4) in the mature stage of the biofilm, bacteria synthesize and release signal molecules; 5) in the bacterial abscission/diffusion stage, bacteria leave the biofilm and return to an independent planktonic lifestyle; 7A2. Expanded conceptual model of biofilm formation; 7B1. The general structure of lipopolysaccharide (LPS), based on that present in *E. coli*; 7B2. The biosynthetic pathway of lipopolysaccharide in *E. coli*. The name of the enzyme is highlighted in red, and the name of the substrate is highlighted in blue; 7C1. The peptidoglycan biosynthetic pathway and sites of action of natural product inhibitors. MurA, known as enol acetone transferase, MurB flavin dependent reductase, MurC, MurD, MurE, and MurF, are four kinds of amino acid ligases, bacterial transposase (MraY), and MurG are transferases responsible for the synthesis of lipid II. 7C2. Antibacterial targets acting on the cell wall. 7D. Purpurin's antibacterial mechanisms.

A biofilm is a special form of bacteria (or fungi) in response to adverse environments. For example, the formation of biofilms increases bacteria resistant to antibiotics.^150^ The process of biofilm formation is involved in many factors,^151^ depending on the strain, nutrient composition, and growth environment. For *P. putida*, the formation of biofilms was mainly controlled by the adhesion protein LapA,^152–154^ while the biofilm formation of *P. aeroginosa* mainly relied on the extracellular polysaccharides Psl and Pel.^155–157^ Traditionally, the formation of biofilms is considered as a five-step model (Fig. 7A1); however, some limitations of this model exist, failing to describe the biofilm complexity^158–160^ from industrial, natural and clinical environments. A dynamic model shown in Fig. 7A2 was later on proposed by K. Sauer.^161^ Up to now, due to limited technologies, it is impossible to track a single cell in the process of biofilm formation, hindering the research on formation of high-resolution biofilms.^162^ As a consequence, the composition required for biofilm formation and the regulation mechanism still remain unknown. Currently, the mechanisms of biofilm resistance include the following points, providing solutions to find possible countermeasures:^163^ 1) antibacterial drug penetration barrier; 2) nutrient restriction; 3) gene phenotype change in the biofilm; 4) QS signal generation; 5) activation of tight response; 6) activation of an efflux pump system; 7) secretion of antibiotic hydrolase, *etc.* In view of the variability and drug resistance of biofilms, modern drugs or strategies for treating bacterial infection caused by biofilm formation are emerging. Among the reported new potential drugs, antibacterial peptides (AMPs),^164^ bacteriophages,^165^ quorum sensing inhibitors (QSIs),^166^ aptamers,^167^ nanoparticles (NPs),^168^ peptide nucleic acids (PNAs),^169^ and anthraquinone-type compounds^170–172^ are attractive and promising solutions.

We herein focus on the anti-biofilm mechanism of anthraquinones. In general, membrane-damaging agents exert the activities through a variety of ways, including the interaction of lipophilic groups and membrane proteins, or the change of the proton dynamics and the inhibition of the electron transfer chain. Anthraquinone and its derivatives as hydrophobic substances are traditionally considered to reduce the hydrophobic interaction between hydrocarbon chains in the phospholipid bilayer, weaken the fluidity of the cell membrane, enhance permeability, and then destroy the biological membrane structure. At the same time, mitochondrial depolarization generating a higher level of reactive oxygen species activates lipid peroxidation and antioxidant defense systems, and the oxidative stress further leads to a significant decrease in the amount of extracellular polymeric matrix and capsular sugars (mannose, xylose and glucuronic acid). This is possibly one of the important anti-MRSA mechanisms of anthraquinone.^173–175^ Biofilm formation is affected by many factors, such as the quorum sensing signal system and a variety of regulating protein genes. The biofilm formation of *Staphylococcus aureus* inhibited by emodin is achieved by blocking cell adhesion. Polysaccharide intercellular adhesion (PIA) is an important component of the *Staphylococcus aureus* biofilm. Synthesis of PIA and the expression of *ica* genes dominate the biofilm formation ability.^176^*Ica* is composed of *icaA*, *icaB*, *icaC* and *icaD*. *IcaA* and *icaD* are central to the PIA generation,^177^ and emodin can reduce the expression of *ica* genes. Besides, emodin stimulation leads to the reduction of DNA polymerases III and IV,^178,179^ and affects gene repair and bacterial resistance. Simultaneous reduction of DNA polymerase III can change bacteria from virulent forms to quiescent ones.^180^ The decrease in the biofilm formation^179,181^ may be caused by the down-regulation of two-component signal transduction systems (TCSs) affecting the autolytic activity and QS. In addition to gene regulation, the anti-biofilm activity of anthraquinone may be related to the polysaccharide of the bacterial complex capsule, ascribed to the biofilm formation of this yeast complex as a capsule-dependent event.^182^ Release of glucuronic xylan (GXM) from the capsule is blocked by interaction of the anthraquinone and capsule, thus affecting the adhesion of yeast cells to the surface and the formation of the extracellular polymer matrix.^183^ Therefore, we infer the anti-biofilm mechanism of anthraquinone shown in Fig. 7D.

#### 3.1.2 Anti endotoxin

The main chemical component of bacterial endotoxin, discovered at the end of the 19th century, is lipopolysaccharide (LPS). Gram negative bacteria have two different membranes, an inner membrane and an outer membrane. LPS distributed in the outer membrane, shown in Fig. 7B2, is toxic.^184^ As the main component of the outer membrane, LPS is crucial to survival of most Gram-negative bacteria. LPS includes three parts: lipid A, a core polysaccharide and an antigen repeating sequence. Lipid A represents the hydrophobic component of LPS located on the surface of the outer membrane, while the core polysaccharide and antigen repeating sequence reside in the surface of bacterial cells.^185,186^ Lipid A is believed to be responsible for the toxic effect of Gram-negative bacteria.^187^ The structure of LPS responsible for the virulence of bacteria varies from bacteria to bacteria.^188^ Accordingly, the enzymes involved in the biosynthesis and transportation of lipid A or LPS are the promising targets for developing new antibiotics. As shown in Fig. 7B1, the purification and characterization of the first three enzymes, LpxA, LpxC and LpxD residing in the lipid A biosynthesis pathway, have been accomplished,^189–191^ providing the structural information of these proteins for designing and developing new antibiotics,^192,193^*e.g.*, to modify the structure of lipid A, to develop new LPS antagonists, or to improve the traditional Gram-negative bacteria vaccine.^194–196^ Unfortunately, although it has been reported that anthraquinones can inhibit bacteria growth *via* blocking the biofunctions of LPS, the mechanisms are poorly studied. The release of endotoxin from *E. coli*^197^ is reduced by the methanol extract of rhubarb. Moreover, the greater the volume fraction of aloe containing serum is, the less the endotoxin residue occurs, indicating that aloe has an inhibitory effect on endotoxin.^198^ Taken together, the antiendotoxin of anthraquinones representing the new antibacterial mechanism deserves to be explored in future research.

#### 3.1.3 Destruction of the bacterial cell wall

Anthraquinones can disrupt the integrity of the bacterial cell membrane and cell wall to achieve their bactericidal activity. They mainly behave in the following two aspects, on the one hand, the structural integrity of the cell wall and cell membrane is destroyed to cause intracellular material outflow, reduction of various intracellular bioactive components, and synthesis or functional impairment of nucleic acids, proteins, ATP, *etc.*; on the other hand, the absorption of nutrients, the excretion of metabolic wastes, the active transport, the passive transport, and the transmission of information rely completely on the cell wall and cell membrane. Although anthraquinones able to disrupt the cell wall are proposed by many papers, the specific targets and action mechanisms have not been systematically reviewed. Therefore, we herein analyze and summarize them on the basis of the previous related results.

The cell wall of Gram-negative and -positive bacteria is mainly reticulated balloons formed by peptidoglycans, *i.e.* high-strength reticulated scaffold structures formed by alternating *N*-acetylcytidylic acid and *N*-acetylglucosamine linked by β-1,4 glycosidic bonds.^199^ It mainly consists of lipopolysaccharide (LPS), peptidoglycan (PG), lipid A-associated protein (LAP), surface-associated material (SAM), phosphopeptidic acid (TA), and other active components.^200^ Basically, the peptidoglycan skeletons of different bacterial cell walls are identical, mainly differing in the composition of amino acids in the tetrapeptide tails and the cross-linking way. As shown in Fig. 7C1, the synthesis of peptidoglycan occurs in three stages at three different bacterial locations.^201^ Since intact peptidoglycan is essential for bacterial survival, all the proteins responsible for cell wall synthesis and regulation are considered important targets in the discovery of new antibacterial drugs.^202^ To date, there are 5 antimicrobial targets reported to participate in the synthesis of the cell wall: 1. enzymes in the synthesis pathway of peptidoglycan,^203–206^ such as MurA–MurG, transglycosylase, and transpeptidase; 2. scaffolding proteins,^207^ including FtsZ protein that mediates bacterial cells to produce Z-loop and regulate cell division, GpsB protein that regulates cell division, DivIVA protein that regulates cell division and sporulation, and EzrA protein that acts in conjunction with GpsB protein to regulate cell wall synthesis; 3. transcriptional regulatory factor, upon exposure to pressure response, bacteria use *σ* gene expression levels regulated by two-component systems (TCSs) and transcription regulators. The bacteria with the *airSR* gene to be knocked out had autolysis, and the gene could directly combine with other genes (*cap*, *pbp1*, *ddl*, *etc.*) to regulate cell wall metabolism;^208^ 4. post transcriptional modification, SRNA regulates the cell wall, for instance, in *Listeria monocytogenes*,^209,210^ the protein Lmo0514 related to the cell wall synthesis recognizes the structure of the classified protease LPXTG, which can covalently connect itself to the cell wall; 5. protein–protein interactions affecting cell wall synthesis and hydrolysis. The dynamic flow of peptidoglycan synthesis and degradation is the main factor responsible for the morphology of bacterial cells. The proteins MreC and MreD are related to peptidoglycan synthesis, and penicillin binding proteins (PBPs) are referred to as peptidoglycan synthetases.^211^ PBPs synthesize cell walls as the main members of the peptidoglycan synthetase system. Penicillin targeting PBPs can inactivate their enzymatic activity, leading to the disorder of the peptidoglycan metabolic flow and thus eliciting the bacteria death.^212^ In addition to peptidoglycan synthetases, the hydrolase activity is crucial for the regulation of peptidoglycan growth, cell division and bacterial morphological changes.

At present, there are two types of antibiotics widely used to inhibit cell wall synthesis: (1) fosfomycin that inhibits the production of the disaccharide oligopeptide precursor in the cytoplasm;^213^ (2) β-lactams that have inhibitory effects on connexin PBPs to block cell wall assembly.^214^ However, some multidrug-resistant bacteria appear insensitive to β-lactams. In the face of antibiotics that can damage the synthesis of the bacterial cell wall, including penicillin and cephalosporin, MRSA escaped the threat of antibiotics by thickening the cell wall *via* enzyme mutant and hydrolase generation.^215,216^ MRSA also has other drug-resistant mechanisms where the decreased sensitivity of MRSA to antibiotics was achieved by the change of cell wall components.^217^ Emodin increased the ability to eliminate drug resistance of *S. aureus in vitro* and *in vivo*, and the antibacterial effect of emodin is the same as that of linezolid, and is superior to that of imipenem, cefepime and other antibiotics.^218^ Using scanning electron microscopy and transmission electron microscopy, the activity of emodin is demonstrated to be closely related to its disruption of the bacterial cell wall and cell membrane integrity.^224^ Treated with emodin, the cell wall and cell membrane became thick and cracked, resulting in the loss of intracellular components. According to the time growth curve, emodin exhibits a time-dependent reduction of bacteria, and the MBC/MIC values of emodin are mostly in the range of 1–2 μM,^219^ suggesting the antibacterial mode belonging to a bactericide function. Exposed to a long dosing of emodin, the MIC of bacteria tested fail to increase.^220^ Moreover, emodin has low toxicity to normal cells, presenting a good safety profile within the range of effective bactericidal concentration.^221–223^ In addition to direct observation means such as electron microscopy, the conductivity of the cell wall and cell membrane is another evaluation approach. When bacteria are treated with anthraquinone derivatives, the conductivity increases significantly,^225,226^ accompanied with the leakage of cell contents, an indication that anthraquinone derivatives can change the permeability of the cell wall. For instance, purpurin^227^ inhibited bacterial growth by interfering with the assembly of the Z ring in the middle of the cell, but not affecting the nucleoid separation, hinting its selectivity to FtsZ. The inhibitory effect of purpurin on mammalian cells is weaker than that on bacterial cells, emphasizing that the antibacterial target of anthraquinones may be FtsZ as shown in Fig. 7C2. In recent years, the scaffold protein FtsZ regulating cell wall division is demonstrated to be a promising target, and FtsZ inhibitors are mainly natural products, small molecular peptides and nucleic acids.

#### 3.1.4 Inhibiting protein synthesis and nucleic acids

Anthraquinones also exert their antibacterial activity by inhibiting corresponding proteins or nucleic acids. Aloe emodin attenuated *S. aureus* pathogenicity by interfering with the oligomerization of α-toxin.^228^ The strategy of targeting virulence factors^229,230^ may give us some inspiration in the design and development of antibacterial drugs. As we mentioned above, rhein can reduce the pathogenicity of *Pseudomonas gingivalis* by reducing the transcriptional genes encoding important virulence factors.^231,232^ Anthraquinones inhibited cell function by penetrating the cell membrane binding with DNA, leading to cell death.^233^ This was supported by Ankita's study^234,235^ that anthraquinones extracted from aloe could inhibit nucleic acid synthesis of *Bacillus subtilis*, affect DNA replication and transcription, and block the protein expression. It has also been found that^236^ rhein can inhibit some oxygen respiration and fermentation genes of *S. aureus* and genes of the ribonucleic acid reductase system, achieving its bacteriostasis. Moreover, anthraquinones can be used as an inhibitor of QS,^237^ preventing the *agr* signal transmission of the *agr* allele of *S. aureus*. Purpurin and quinalizarin can inhibit the expression of the *hla* gene that plays an important role in the biofilm formation.^238^ Additionally, various reports on the antibacterial effects of anthraquinones *via* protein inhibition have been published. Emodin inhibited the growth of *Haematococcus parasuis* by suppressing the expression of key proteins distributed in the ribosome synthesis, ABC transport system, carbohydrate metabolism pathway and bacterial cell division.^239^ Anthraquinones are discovered to inhibit FtsZ protein,^240^ and interfere with the activity of the pyruvate pathway, and inhibit ribosome proteins and the aminoacyl tRNA synthetase of MRSA.^241^

Purpurin inhibits biofilm-related genes (*spa*, *psmα* and *rbf*) and the α-hemolysin *hla* gene and controls the expression of the *cid*/*lrg* gene. In another study, purpurin can inhibit the growth of Gram-negative and -positive bacteria producing *O*-acetylated peptidoglycan and APE with IC_50_ values ranging from 0.3 to 23 μM.^242,243^ Purpurin displayed antibacterial activity against 24 strains of 6 *Candida* species with MICs ranging from 1.28 to 5.12 μg mL^−1^. Anti-bacterial mechanisms showed that purpurin induced apoptosis of *Candida* cells through depolarizing mitochondrial membrane potentials, one of the biochemical checkpoints controlling cell death in eukaryotic cells, and formed biofilm and mycelium by blocking an energy dependent efflux pump.^244–247^ The mechanisms are presented in Fig. 7D.

#### 3.1.5 Inhibition of bacteria respiratory metabolism

Respiratory metabolism is a main way for organisms to obtain required energy for life activity, including the tricarboxylic acid cycle, glycolysis, and pentose phosphate. The respiratory metabolic process of microorganisms is inhibited, leading to the reduced generation of energy and the carbon skeleton in the metabolic activity, and thus affecting the normal growth and reproduction of microorganisms. The microorganism growth inhibited by antibiotics was proved to be related to the suppression of cell respiration,^248^ while the cell death caused by most bactericidal antibiotics was associated with the acceleration of respiration. Knockout of cytochrome oxidases inhibiting cellular respiration is sufficient to attenuate bactericidal lethality, whereas acceleration of basal respiration by genetically uncoupling ATP synthesis from electron transport chains results in potentiation in the killing effect of bactericidal antibiotics. Anthraquinones reduce the respiratory control index and P/O value (the relationship of ATP synthesis and oxygen consumption) of rat liver mitochondria through the uncoupling mode and enhance the antibacterial effects.^249^ Also, the anthraquinones from rhubarb exhibit anti-coliform activities *via* inhibiting electron transfer and decoupling effects.^250^ Emodin has a potential inhibitory effect on a variety of human liver cancer cell lines, stimulating the expression of *p53* and *p21* genes to inhibit respiration and arrest the cell cycle.^251,252^ Besides, the key enzymes are inhibited by anthraquinones in the tricarboxylic acid cycle and cell energy metabolism of eukaryotic cells and prokaryotic cells.^253,254^ For example, blockage of SDH (successive dehydrogenase) and MDH (malate dehydrogenase) by anthraquinones can significantly inhibit the respiration of *Staphylococcus aureus*, accounting for a respiratory inhibition rate of 40%.

#### 3.1.6 Inhibiting other substances

Anthraquinones has other antibacterial mechanisms, including the regulation of efflux pumps, enzymes, and active oxygen species. The antibacterial activity of rhein is realized by regulating the enzyme in microorganisms,^255^ as the concentration increases, the *N*-acetyltransferase activity of *Helicobacter pylori* decreases, and then the nucleic acid synthesis is inhibited accordingly. Anthraquinones improve the inhibitory activity of efflux pumps, along with low activity against some multidrug-resistant bacteria.^256,257^ A convincing example is taken that emodin has poor antibacterial activity against *Escherichia coli*, but is resistant to PaβN (outflow pump inhibitor) and significantly increases the antibacterial activity of other antibiotics, indicating that the antibacterial mechanism of emodin is possibly associated with regulating the activity of outflow pumps.^258^ In addition, some anthraquinone derivatives generating reactive oxygen species (ROS)^259–262^ with photosensitization, especially singlet molecular oxygen (^1^O_2_), superoxide anions, and hydroxyl radicals, produce oxidative damage to cause physiological reactions in bacteria,^263–265^ thus achieving bactericidal effects.

Besides, according to recent reports, good inhibitory effects of anthraquinone-type derivatives on fungi have been observed. In terms of mechanism, it not only can induce apoptosis of fungal mitochondria by depolarizing the membrane potential, and but also can inhibit the function of efflux pumps.^266^ Anthraquinone derivatives restrict *C. dubliniensis* biofilm production in a concentration-dependent manner; this was supported by mature biofilms less susceptible to purpurin. Their MMP-independent apoptosis is triggered by the increased intracellular ROS levels in fungal biofilms and MMP depolarization, followed by DNA degradation. In the *C. albicans* biofilm under hypha-inducing conditions, anthraquinone derivatives block the yeast-to-hypha transition followed by the distortion of biofilm synthesis, resulting in decreased metabolic activities. Anthraquinone derivatives reduce the expression of hypha-specific genes including ALS3, HYR1, HWP1, and the hyphal regulator RAS1.^267,268^

### 3.2 Toxicity of anthraquinones

Safety is a prerequisite for a therapeutic drug. Although anthraquinone drugs are reported to have no toxic and side effects when used reasonably, the antibacterial activity of anthraquinone is related to its toxicity. The toxicity of some anthraquinone derivatives is undeniable, in spite of the unconfirmed correlation of the long-term use and cancer induction. Cumulative anthracyclines such as doxorubicin result in cardiotoxicity strongly associated with redox cycling and generation of free radicals, primarily limiting clinical applications. The toxicity and mutagenicity of hydroxyanthraquinones used as laxative agents have been demonstrated *in vitro* and *in vivo*. The main toxicity of anthraquinones comes from the ability to act as a Michael receptor interacting with some nucleophilic reagents in cells, such as NADPH, producing toxic substances to damage cells. The redox reaction generates some superoxide radical anions, but the occurrence of oxidation–reduction is influenced by environmental factors including oxygen and pH.^269,270^ The interaction of anthraquinones with some nucleophilic reagents may also produce thiols that interfere with the regulation of normal cells. Noticeably, anthraquinone derivatives are usually not prone to the Michael addition reaction due to the quinone positions α and β blocked by two benzene rings.^271^ Moreover, the role^269–271^ of anthraquinone as an oxidant or a reducing agent in the *in vivo* redox process remains to be identified. Accordingly, despite the large body of evidence on the involvement of anthracyclines in redox reactions, the exact degree of contribution to the antibacterial activity and toxicity in the clinic remains to be explored.

## 4. Concluding remarks and perspectives

In this review, the structure–activity relationships of anthraquinone and its derivatives are summarized in detail in Fig. 2 and 4–6 for the first time, and the antibacterial mechanisms and toxicity of anthraquinones are systematically analyzed. From analysis of structure–activity relationships, some conclusions are reached that the hydroxyl groups on the anthraquinone ring relate to a variety of pharmacological activities, including antibacterial, anti-cancer, and anti-inflammatory, and the polarity of substituents on anthraquinones obviously affects the antibacterial activity of anthraquinones, and the stronger the polarity is, the more potent the antibacterial effect is, and the existence of hydroxyl groups is not necessary in the antibacterial activity of hydrogenated anthraquinone derivatives. Anthraquinones have obvious antibacterial effects on many clinical drug resistant bacteria. However, most of the studies on the antibacterial activity of anthraquinone derivatives against microorganisms remain on *in vitro* evaluation, and relatively few *in vivo* tests of antibacterial anthraquinones are reported. Therefore, an appropriate experimental method selected and *in vivo* and *in vitro* comprehensive efficacy evaluation are vital to obtain antibacterial anthraquinone-based agents with better pharmacokinetics and more potent efficacy.^272^

The structure determines the function and action mechanism. A rigid planar structure is necessary for the antibacterial activity. The skeleton structure of the benzene ring in hydroxy-anthraquinones, as a core part quinone responsible for the biological activity, affects DNA biology. The replacement of the benzene ring in anthraquinone with an aliphatic ring has no significant effects on bacterial DNA biology. The presence of electron-rich substituents seems to be more conducive to the biofilm inhibition; the hypothesis was confirmed by the decreased activity due to an isopentane substituent, and a targeted elimination ability of an isopentene group to biofilms, along with no effect on the antibacterial mechanism in the presence of electron-deficient substituents. Noticeably, the antibacterial effects of anthraquinone glycosides are accomplished by mainly inhibiting corresponding enzymes or proteins, and the improved water solubility.

It is noted that the long-term and extensive abuse of any drug inevitably leads to drug resistance, thus reasonable drug use and drug combination are optimal options. There are many reasons causing drug resistance, anthraquinone drugs are no exception for resistance development. The significantly reduced susceptibility of fluoroquinolones to bacteria occurs in clinics due to the corresponding gene mutations in pathogens. Tetracycline as an anthraquinone-type drug inhibits protein synthesis by interfering with the 30S subunit of ribosomes, and its drug resistance mechanism is usually involved in the active removal from bacterial cells by outflux pumps, and no effect due to ribosome protection. The antibacterial mechanisms of anthraquinone derivatives reported vary a lot, and biofilm formation of outside bacteria is inhibited and eliminated by anthraquinone derivatives, and they also can interact with genes and proteins inside bacteria, demonstrating an advantage of multi-target antibacterial mechanisms. However, the drug resistance development of anthraquinone derivatives is reported to have a close relationship with the production of inactivated enzymes and changes in their target sites. Meanwhile, anthraquinone derivatives exert the elimination effects of other multi-drug resistance. A good example is illustrated where 87 reverses multidrug resistance by weakening the function of the ABCG2 transporter causing multidrug resistance.^273^

In other words, anthraquinone derivatives widely distributed in the plant kingdom exhibit significant pharmacological effects and broad market prospects. Our future research focuses might as well be shifted according to the pharmacological activities. Identification of structural similarities between natural product structures and protein sub-folding are a powerful tool for developing natural product-derived drugs.^274,275^ More importantly, the development of traditional Chinese medicine or traditional Chinese medicine preparations containing anthraquinones in combating drug resistant bacteria has great application prospects.

## Conflicts of interest

The authors confirm that this review article has no conflicts of interest.

## Supplementary Material
